# Efficacy of a joint supplement containing eggshell membrane among other ingredients to improve the mobility of dogs with osteoarthritis: a multicenter double-blind randomized placebo-controlled study

**DOI:** 10.3389/fvets.2025.1561793

**Published:** 2025-06-02

**Authors:** Guillaume R. Ragetly, Ângela Martins, Ciprian A. Ober, Silvia Boiocchi, Céline S. Nicolas

**Affiliations:** ^1^Centre Hospitalier Vétérinaire Frégis, Gentilly, France; ^2^Faculty of Veterinary Medicine, Lusofona University, Lisbon, Portugal; ^3^Superior School of Health, Protection and Animal Welfare, Polytechnic Institute of Lusophony, Lisbon, Portugal; ^4^Arrábida Veterinary Hospital, Arrábida Animal Rehabilitation Center, Azeitão, Portugal; ^5^Department of Surgery and Intensive Care, Faculty of Veterinary Medicine, University of Agricultural Sciences and Veterinary Medicine, Cluj-Napoca, Romania; ^6^Clinica Veterinaria Vezzoni, Cremona, Italy; ^7^Virbac SA, MU Petfood Petcare, Carros, France

**Keywords:** mobility issues, joint disorder, nutraceutical, canine osteoarthritis, Movoflex, pain, movement, mobility improvement

## Abstract

The management of osteoarthritis (OA) in dogs is typically multimodal, including weight management, activity adjustment, joint supplements, and medical treatments when needed. This study evaluated the efficacy of a joint supplement containing eggshell membrane, krill meal with omega-3 fatty acids, *Haematococcus pluvialis* as a source of astaxanthin, hyaluronic acid and a *Boswellia Serrata* extract, in dogs with OA, in a multicenter, randomized, placebo-controlled trial. Fifty-two dogs with confirmed OA were given the test supplement or a placebo, for 90 days. Owners regularly completed two validated questionnaires for osteoarthritis (CBPI and LOAD) and rated their dog’s discomfort every 15 to 30 days. Monthly evaluations by investigators included assessments of the dog’s posture, gait, joint pain upon palpation and range of motion (from 1-normal to 4-severe or severely impacted) to determine a clinical score. Statistical analyses included both within-group and between-group comparisons. Of the 52 dogs enrolled, 46 completed the study, with 22 receiving the supplement and 24 receiving the placebo. All main parameters significantly improved over time in the supplement group (CBPI pain severity, CBPI pain interference, LOAD, discomfort, clinical score). In the placebo group, only the CBPI pain interference and LOAD improved. However, there was a statistically significant difference between groups for the CBPI pain interference (*p* = 0.009). Therefore, this study demonstrates that the test supplement can improve the mobility and quality of life of osteoarthritic dogs.

## 1 Introduction

Osteoarthritis (OA) is a chronic inflammatory disease impacting the entire joint (1). It is one of the most diagnosed diseases in dogs (2, 3). The prevalence of OA can range from 2.5% in a dog population under primary veterinary care to more than 80% of older and obese dogs (2–4). Although OA can be present in young dogs, age, body weight, breed and neuter status have been identified as risk factors (5–8). The most commonly affected joints are elbows, hips, tarsus, shoulders and stifles (8, 9).

Clinically, the structural and functional changes in the joint will lead to pain, inflammation and an altered mobility. This will translate into a change in gait and weight distribution, the presence of lameness, pain upon palpation, and decreased joint range of movement (10, 11). Owners will describe their dogs as being stiff (with variability throughout the day), having difficulty to perform certain activities (like walking or running, jumping, or even playing) or being reluctant to exercise (12, 13). A change of behavior or demeanor is also usually observed and described by owners (12, 13). Radiographic changes like osteophytes, subchondral bone sclerosis and joint effusion can be observed and used as a diagnostic tool (14). Overall, the diagnosis of OA can be based on objective and subjective measures like clinical metrology instruments, gait assessment and radiography. Clinical metrology instruments (CMIs) are validated questionnaires filled in by owners to evaluate and address clinically relevant questions about a specific construct (13). There are several validated CMIs for OA, including the Canine Brief Pain Inventory (CBPI) and Liverpool Osteoarthritis in Dogs index (LOAD), which are commonly used (13, 15–17).

The therapeutic goals center on alleviating joint pain and enhancing motor function to improve the quality of life of the affected animals. Based on the American Animal Hospital Association (AAHA) guidelines for pain management, a multimodal approach is necessary to improve dogs affected by OA (18). This approach includes a nutritional management to control the dog’s body weight with a therapeutic diet rich in omega-3 fatty acids (FA) to also limit the inflammation, an adaptation of the dog’s exercise and environment to limit high impact activities while still facilitating movements, and a physical rehabilitation program (at home and in specialized centers) to maintain the dog’s mobility (1, 11, 18–21). When needed, effective analgesics may be used. According to the AAHA guidelines, medications that seem the most effective are non-steroidal anti-inflammatory drugs (NSAIDs) and, potentially, anti-nerve growth factor monoclonal antibodies (anti-NGF) (18). Adjunctive analgesic therapies (like amantadine, gabapentin, acetaminophen, steroids or tramadol) may also be considered if necessary (1, 18).

The need for frequent administration of NSAIDs or other pain medicines, along with their side effects and burden of care on the owner, necessitates the use of alternative therapies (22, 23). Among alternatives, nutraceuticals are a good option as they are usually safe. In fact, regardless of the OA severity, efficient joint supplements could still help maintain healthy parts of joints and slow down their degradation (1, 24). However, joint supplements are not all the same and it was found in a recent meta-analysis that those with omega-3 fatty acids showed evident clinical analgesic efficacy, those with collagen (including eggshell membrane-based supplements) showed only a weak efficacy while those only based on chondroitin sulfate and/or glucosamine had no proven efficacy (20). In the AAHA guidelines for pain management, the omega-3-based supplements are considered the most efficacious, based on evidence-based veterinary medicine while those not based on omega-3 FA are considered only as adjuncts, with fewer or no demonstrated efficacy (18).

The test supplement is a joint supplement with a mix of five key ingredients of natural origin, independently known to improve joint health and mobility in dogs or to have antioxidant effects. It contains eggshell membrane (ESM), a complex ingredient full of different molecules naturally found in joints, including collagen, glucosamine, glycosaminoglycans, elastin, hyaluronic acid, and other proteins and amino acids (mainly proline, glutamic acid, and glycine) that can help support protein synthesis (25, 26). The eggshell membrane used in the test supplement has proven efficacy in humans and dogs with mobility disorders (27–29). Other studies, in humans and dogs, with different types of ESM have also shown the beneficial effects of this ingredient in joints (30–34).

The supplement also contains hyaluronic acid (HA) of different molecular weights (MW, below 50 kDa and above 1 MDa). Indeed, the action of HA can depend on its MW, with higher MW being mostly involved in the lubrication and viscoelasticity of the synovial fluid and resilience of the cartilage, while lower MW HA could help initiate the restorative processes (35, 36). Although the effect of HA has mainly been investigated when injected (but not only), the oral bioavailability of HA and distribution in joints has been demonstrated in dogs, even with high molecular weight HA (37).

*Haematococcus pluvialis*, one of five key ingredients, is one of the richest and safest natural sources of astaxanthin, a powerful antioxidant which can be absorbed by dogs when given orally (38–40). Since oxidative stress plays an important role in OA evolution, controlling it with antioxidants may help delay joint degradation (41).

Krill has also been added as a source of omega-3 fatty acids (FA) that come in the form of readily absorbed phospholipids (42, 43). It is a better source of omega-3 FA than fish oil, based on the omega-3 index observed in dogs fed fish oil or krill meal (43, 44). Krill is effective in improving joint health in animal models and humans and in supporting active dogs (45–49). The phospholipids in krill can also facilitate the absorption of astaxanthin and HA (50–52), providing a synergistic effect with these ingredients. Supplements combining krill, astaxanthin and HA have shown promising results in human and animal models of OA, modulating the inflammatory cascade, reducing cartilage degradation and improving pain and joint function (53–55).

Finally, *the test supplement also contains a Boswellia serrata* extract. It is rich in boswellic acids known to modulate inflammatory processes, and has been shown to have beneficial effects on joints and mobility, including in dogs (56–58). The bioavailability of the boswellic acids has only been assessed in other species than dogs (56). They are known to be lipophilic and their bioavailability might therefore be increased by the addition of lipids (56), like the krill phospholipids.

When the test supplement was given to owners of dogs with mobility issues for two months, the general mobility and other mobility parameters assessed by the owners significantly improved, with some improvements observed as early as day 7 (59). Similar formulations of this ESM-based supplement have also demonstrated good effectiveness in dogs with mobility disorders, including in dogs with OA in a pilot clinical study (60, 61). However, a blinded placebo-controlled clinical trial involving veterinarians and a sufficient number of dogs still had to be performed to evaluate the efficacy of this formula.

The objective of the study presented here is to evaluate the efficacy of a supplement containing the five main ingredients described above in improving the mobility of dogs with OA, in a blind placebo-controlled clinical trial.

## 2 Materials and methods

This clinical study was a multicenter double-blind randomized placebo-controlled trial performed on client-owned dogs with confirmed OA. It was approved by the Virbac Ethical Review Committee prior to the start of the study (Approbation number; EU-ERC 2022008–02). The protocol complied with European Directive 2010-63-EU and application of the 3Rs principles and Virbac Code of Animal Care. The study took place in nine specialized veterinary clinics in seven European countries (Hungary, Romania, Latvia, Portugal, Spain, Italy, and France) between January 2023 and February 2024. All owners gave their consent to participate in the study.

### 2.1 Animals

Client-owned dogs with signs of mobility issues were recruited based on the following inclusion criteria: at least 3 years of age; body weight between 15 and 35 kg; mobility disorders present for at least 3 months, according to the owner; presenting at least two signs of mobility issues (difficulty to walk / lag behind during walks; difficulty to stand up after lying down; difficulty to jump; difficulty to walk up or down stairs; clear lameness or stiffness after exercise); and radiographic evidence of OA, with definite osteophytes, in at least the hip, elbow, carpal or tarsal joint. Exclusion criteria included any concomitant systemic or neurological disease such as cardiovascular disease, immune-mediated disease (e.g., lupus), obesity (body condition score >7/9 or 4/5), infection, neoplastic disease, or allergies. Pregnant or lactating bitches were also excluded. Dogs who received NSAIDs, glucocorticoids or analgesics recently were excluded unless a wash-out period was respected: 2 weeks for NSAIDs, ‘short-acting’ glucocorticoids (e.g., oral prednisone, and topical glucocorticoid preparations), gabapentin or tramadol; 2 months for oral or parenteral ‘long-acting’ glucocorticoids (e.g., injectable methylprednisolone acetate) and anti-NGF monoclonal antibodies; 3 months for intra-articular injection of any material into any joint. Dogs with any joint instability due to ruptured ligament, dogs with stifle disease, and dogs who had surgery on any joint within 180 days before enrollment were excluded. Indeed, short-term progression (6 months as defined by Cook et al. (62)) could be constant after surgery and could introduce a bias. Dogs with stifle OA were excluded to avoid bias, as their lameness could stem from post-operative complications, mechanical instability, or meniscal damage rather than OA alone (14, 63). For ethical reasons, acute pain management with analgesic drugs (e.g., gabapentin, tramadol) were authorized in case of severe pain or marked decrease of quality of life (QoL), if stopped at least 1 week before the next visit. All treatments given for pain were noted down and considered rescue treatments. Nutraceuticals were allowed if administered for at least 12 weeks prior to enrollment and maintained during the whole study period.

### 2.2 Products and randomization

The product tested was a joint supplement (Movoflex^Ⓡ^ Soft Chews, Virbac, France), containing eggshell membrane (3.3%), krill meal (1.85%), algae meal (*Haematococcus pluvialis* – 0.26% – as a source of astaxanthin), hyaluronic acid (of high & low molecular weights – 0.49%), and a *Boswellia serrata* extract (0.57%). Other ingredients included pre-gelatinized rice, glycerine, derivatives of vegetable origin, pre-gelatinized maize starch, sunflower refined oil, sorbitol, sugars, pea protein, yeasts, pre-gelatinized rice starch, maltodextrin, minerals, rapeseed oil, and powder cellulose. The placebo (same formula but without the 5 key ingredients listed above: eggshell membrane, krill meal, algae meal, hyaluronic acid and *Boswellia serrata* extract) was a soft chew that matched in size the joint supplement. The supplement and its placebo were in white jars labeled A or B so that both investigators and owners were blinded to the treatment group. Each jar contained 30 chews and three jars were given for the duration of the study. One soft chew per day (test or placebo) adapted to dogs 15–35 kg was given for 90 days. Dogs were randomly allocated to product A or B using a table of random numbers with a block randomization design of size 2.

### 2.3 Design and outcomes measured

After inclusion of an animal in the study on day 0, the owner completed the Canine Brief Pain Inventory (CBPI) and Liverpool Osteoarthritis in Dogs index (LOAD), two validated clinical tools to assess osteoarthritis in dogs (15–17). At the end of the CBPI inventory, the owner must grade the dog’s quality of life from 1-poor to 5-excellent. The owner also had to grade the dog’s discomfort from 1-none to 4-unbearable. Explanations about the questionnaires and guidance for the replies to provide were given to the owners. The same questionnaires were completed after 15, 30, 60, and 90 days by the same owner (except for one dog, on one occasion). The investigator performed a clinical evaluation to grade the dog and affected joints. Measures included evaluation of the effect on the dog’s posture and motion (from 1-normal to 4-severely abnormal) and on the joint’s pain upon palpation (from 1-none to 4-severe) and passive range of movement (ROM – from 1-normal to 4-severely abnormal), as described previously (64). The sum of the four scores given was used as the clinical score (from 4 to 16). This clinical evaluation was also performed after 30, 60 and 90 days in the study.

### 2.4 Statistical analysis

Based on the LOAD data from a previous pilot study performed on a similar formulation, with a mean of 15 in the test group and of 24 in the placebo group on Day 84, and a standard deviation of 9, at least 22 cases per group were required in order to see a statistically significant difference with an *α* of 0.05 and power level of 0.9 (61). The mean dogs’ age and body weight were compared between groups at D0 using a Student’s t-test in case of normal distribution and otherwise using the Mann–Whitney (Wilcoxon) test. A Friedman test was used for intragroup comparisons of the scores over time. In case of significance, *post hoc* pairwise comparisons were performed using Dunn’s multiple comparisons test for comparison of data versus day 0. If a statistical significance was obtained in both groups, a generalized linear mixed-effects model was used for intergroup analyses of the scores over time (with the time as fixed effect and the dog as random effect), focusing on the time x treatment interaction for significance. The use of rescue analgesia and the compliance were compared between groups using Kendall’s Tau b.

The significance level was set at *p* < 0.05. Analyses were performed using the Statgraphics Centurion (version XVI.II) and GraphPad Prism (version 10) softwares.

## 3 Results

### 3.1 Study population

A total of fifty-two dogs were recruited. Twenty-six dogs received the test supplement and twenty-six received the placebo. In the supplement group, one dog abandoned the study after three weeks for a lack of improvement, two had OA on the knee at inclusion (an exclusion criteria) and one was euthanized for a cardiac tumor. These four dogs were therefore removed from the analysis. In the placebo group, one dog was lost to follow-up and one had OA on the knee and were therefore removed from the analysis. In the end, twenty-two dogs in the supplement group and twenty-four dogs in the placebo group were analyzed. The compliance was overall very good in all dogs. A few dogs (eight in each group) missed some administrations in both groups but only occasionally (less than a third of the time) and were therefore kept for analysis. The percentage of non-compliance was not significantly different between groups (*p* > 0.5 at each period). No side effects were reported during the study.

Among the analyzed dogs, fifteen dogs received NSAIDs at some point since being diagnosed with OA. Two were still taking NSAIDs two weeks prior the enrollment and had to apply a wash-out period before starting the study. Two dogs received anti-NGF monoclonal antibodies in the past, including one in the last two months before enrollment. This dog had to apply a wash-out period before starting the study. Some dogs also received joint supplements (*n* = 19) and/or fish oil (*n* = 9) that could be continued if given for at least twelve weeks prior to inclusion and continued throughout the study. Other past therapies included IA injections (*n* = 3), rehabilitation/physiotherapy (*n* = 3), laser and magnetic therapy (*n* = 1, each).

On day 0, there was no difference between the groups for the breed, sex, age, body weight, OA grade (assessed by radiography) or joint(s) affected (Table 1). There were also no significant differences between groups on day 0 for the LOAD, discomfort, clinical score and subscores despite apparent higher scores in the supplement group (Table 2). However, the CBPI scores for pain interference, total, and quality of life were significantly higher (worse) in the supplement group (Table 2).

**Table 1:** Demographic information of enrolled dogs that completed the 3-month study and were included in data analysis.

Characteristic	Supplement (*n* = 22)	Placebo (*n* = 24)
Breed (number – %)		
Crossbreed	7 (32%)	6 (25%)
Labrador retriever	2 (9%)	6 (25%)
German shepherd	4 (18%)	3 (12%)
Golden retriever	2 (9%)	2 (9%)
American Bull Terrier	1 (5%)	1 (5%)
American Staffordshire Terrier	0	2 (8%)
Other breeds	6 (27%)	4 (16%)
Sex (number – %)		
Female	13 (59%)	12 (50%)
Male	9 (41%)	12 (50%)
Age (mean in years – SD)	7.5 (4.4)	6.8 (3.5)
Body weight (mean in kg – SD)	28.2 (6.5)	27.6 (6.1)
OA grade (number – %)		
Grade 2	6 (27%)	9 (37.5%)
Grade 3	10 (46%)	9 (37.5%)
Grade 4	6 (27%)	6 (25%)
Joint(s) affected (alone or combined) (number – %)		
Hip(s)	5 (23%)	7 (29%)
Elbow(s)	13 (59%)	12 (50%)
Tarsal joint(s)	1 (5%)	0
Several joints	3 (14%)	5 (21%)

**Table 2:** Baseline scores of the parameters assessed by owners and veterinarians.

Assessment	Supplement (*n* = 22)	Placebo (*n* = 24)
CBPI		
Pain severity	4.375 (0–8.75)	3.5 (1–6.75)
Pain interference	6.833 (1.167–8.833)	3.333 (0.833–8.667) *
Total	11.375 (1.167–17.083)	6.625 (1.833–15.167) *
Quality of life	2 (1–4)	3 (2–4) *
LOAD	29.5 (6–39)	23 (9–34)
Discomfort	3 (1–4)	2 (1–4)
Clinical score	11 (4–16)	9 (4–14)
Effect on static posture	2.5 (1–4)	2 (1–4)
Effect on motion	3 (1–4)	3 (1–3)
Pain upon palpation	3 (1–4)	2 (1–4)
Passive ROM	3 (1–4)	2 (1–4)

Results are expressed as median (min-max). A lower score means better mobility, except for the quality of life (the higher, the better). ROM, Range of movement; *p < 0.05 between groups.

### 3.2 Evolution of parameters assessed by owners

**Figure 1:**
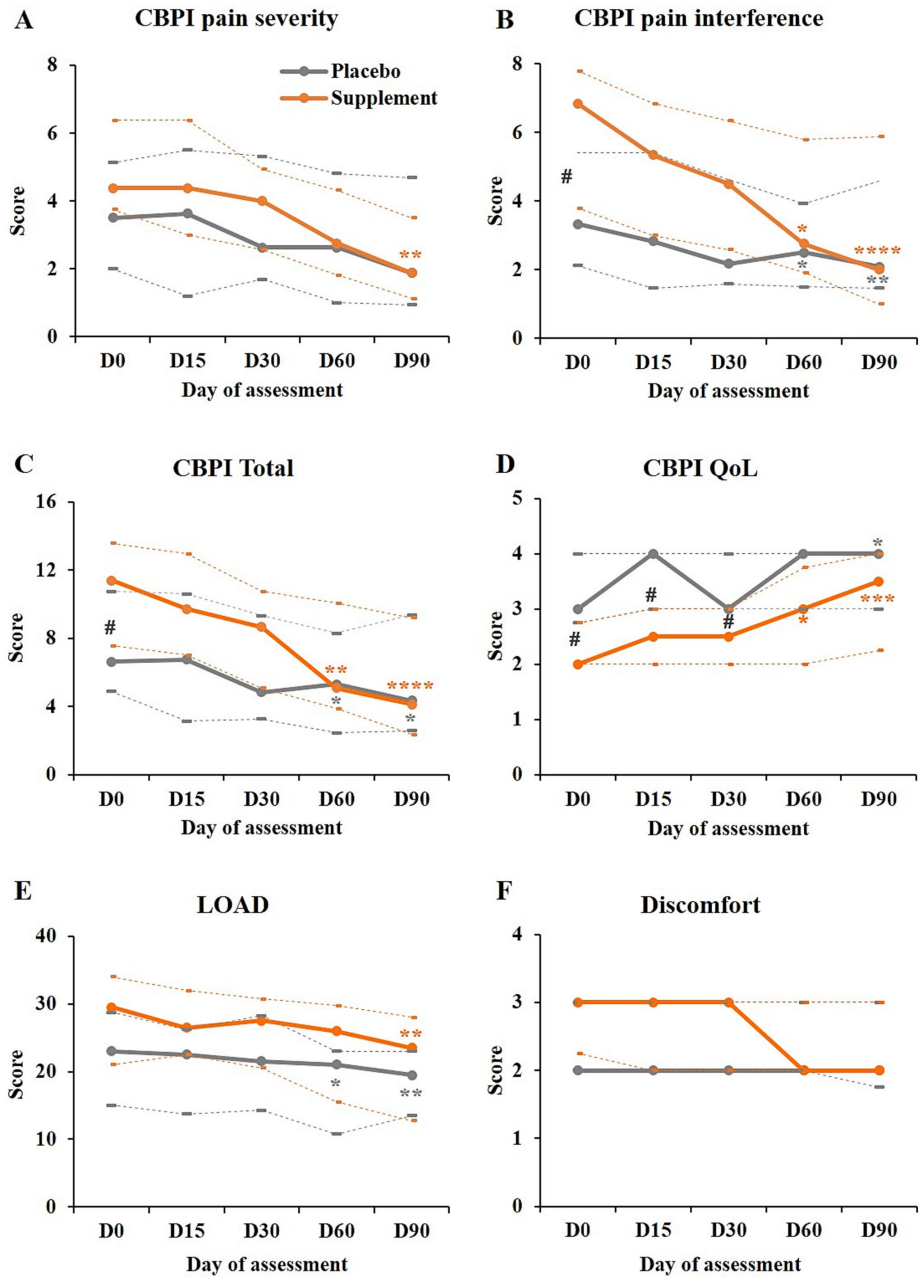
Evolution of the parameters assessed by owners. **(A–D)** Scores obtained for the CBPI pain severity, pain interference, total, and for the quality of life (QoL), as indicated, in the supplement group (orange) and placebo group (grey). **(E,F)** LOAD and discomfort scores obtained in both groups [legend as in **(A)**]. In all graphs, the plain lines represent the medians, and dotted lines below and above represent the first and third quartiles, respectively. In all graphs except the CBPI quality of life, a decrease in score represents an improvement. Pairwise comparisons versus day 0: *p < 0.05; **p < 0.01; ***p < 0.001; ****p < 0.0001. ^#^*p* < 0.05 between groups.

The CBPI pain severity score (PSS) significantly improved over time in the supplement group (−57% in median score by day 90, *p* = 0.0003, Table 3) but not in the placebo group (−46%, *p* = 0.1008, Table 3). Pairwise comparisons versus day 0 showed a significant improvement on day 90 in the supplement group (*p* = 0.0034, Figure 1A; Table 3).

**Table 3:** Evolution of the main parameters over time.

Assessment	Product	Day 0	Day 15	Day 30	Day 60	Day 90	*p*-value^a^
CBPI pain severity	S	4.37 (8.75)	4.37 (7.75)	4.00 (7.25)	2.75 (7.00)	1.87 (7.00) **	**0.0003**
	P	3.50 (5.75)	3,62 (6.75)	2.62 (7.25)	2.62 (8.50)	1.87 (7.75)	0.1008
CBPI pain interference	S	6.83 (7.67)	5.33 (8.83)	4.50 (7.83)	2.75 (7.83) *	2.00 (7.5) ****	**0.00002**
	P	3.33 (7.83)	2.83 (8.00)	2.17 (8.50)	2.50 (9.17) *	2.08 (9.17) **	**0.0018**^**##**^
LOAD	S	29.5 (33)	26.5 (32)	27.5 (31)	26 (30)	23.5 (29) **	**0.0003**
	P	23 (25)	22.5 (26)	21.5 (31)	21 (44) *	19.5 (44) **	**0.0051**
Discomfort	S	3 (3)	3 (3)	3 (3)	2 (3)	2 (3)	**0.0083**
	P	2 (3)	2 (3)	2 (3)	2 (3)	2 (3)	0.0554
Clinical score	S	11 (12)		11 (10)	9.5 (10) *	9.5 (10) **	**0.00001**
	P	9 (10)		8 (9)	8 (10)	8 (11)	0.0692

S, Supplement; P, Placebo. Results are expressed as median (range). A lower score means better mobility, except for the quality of life (the higher, the better). ^a^*p*-value from the Friedman test. Bold *p*-values depict a significant difference over time (*p* < 0.05). *p < 0.05; **p < 0.01; ***p < 0.001;****p < 0.0001 vs day 0 in the same group. ^##^*p* < 0.01 when comparing groups between them.

The CBPI pain interference score (PIS) significantly improved over time in both groups: −71% in median score by day 90 in the supplement group (*p* < 0.0001) and −38% in the placebo group (*p* = 0.0018, Table 3). However, the improvement over time was significantly greater with the supplement than with the placebo (*p* = 0.009). Pairwise comparisons versus day 0 showed a significant improvement on days 60 and 90 in the supplement group (*p* = 0.0304 and *p* < 0.0001, respectively) and in the placebo group (*p* = 0.0139 and *p* = 0.001, respectively, Figure 1B; Table 3).

The total CBPI score (sum of both previous scores) significantly improved over time in both groups: −64% in median score by day 90 in the supplement group (*p* < 0.0001, Supplementary Table 1) and −35% in the placebo group (*p* = 0.0186, Supplementary Table 1). The improvement over time was also significantly greater with the supplement than with the placebo (*p* = 0.0187, Supplementary Table 1). The improvement versus day 0 was significant as of day 60 in the supplement group (*p* = 0.0048 and *p* < 0.0001 on days 60 and 90, respectively) and in the placebo group (*p* = 0.0423 and *p* = 0.0104, respectively, Figure 1C; Supplementary Table 1).

The quality of life in the supplement group was significantly worse (lower score) than in the placebo group on days 0 to 30 (*p* < 0.05, Figure 1D); by day 60, there was no more difference between groups. While both groups showed a significant improvement over time (*p* < 0.0001 and *p* = 0.0071 in the supplement and placebo groups, respectively, Supplementary Table 1), with no significant difference between groups, the improvement in median score by day 90 was only of 33% in the placebo group but of 75% in the supplement group. Pairwise comparisons versus day 0 showed a significant improvement on days 60 and 90 in the supplement group (*p* = 0.0145 and *p* = 0.0005, respectively) and on day 90 in the placebo group (*p* = 0.0371, Figure 1D; Supplementary Table 1).

The LOAD score significantly improved over time in both groups (*p* = 0.0003 and *p* = 0.0051 in the supplement and placebo groups, respectively, Table 3). The median scores decreased by 20% in the supplement group and by 15% in the placebo group by day 90, with no significant difference between groups. Pairwise comparisons versus day 0 showed a significant improvement on day 90 in the supplement group (*p* = 0.0017) and on days 60 and 90 in the placebo group (*p* = 0.0186 and *p* = 0.0029, Figure 1E; Table 3).

Consistently with the other parameters, the discomfort in the supplement group seemed to be more severe than in the placebo group on day 0, although not to a significant level (median of 3 vs. 2, respectively, Table 1; Figure 1F). Over time, the dog’s discomfort significantly improved in the supplement group (−1 point in median score or 33% change by day 90; *p* = 0.0083) but not in the placebo group (no change in median score; *p* = 0.0554, Figure 1F; Table 3). However, pairwise comparisons with day 0 did not show any significant difference in the supplement group (Figure 1F; Table 3).

### 3.3 Evolution of parameters assessed by investigators

**Figure 2:**
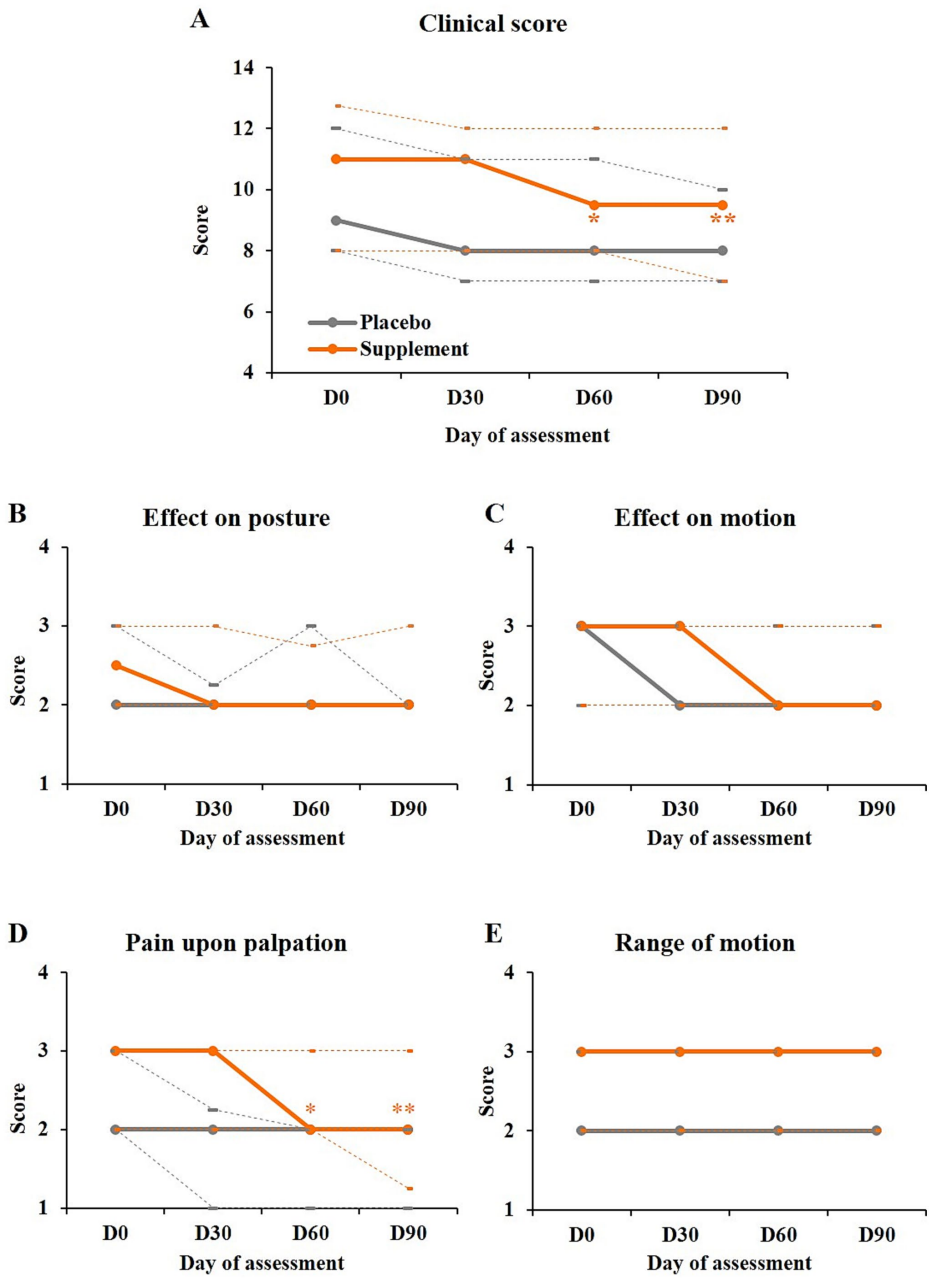
Evolution of the parameters assessed by the investigators. **(A)** Clinical scores (sum of the four subscores) obtained in both groups. **(B–E)** Subscores of the clinical score as indicated. Legend as in Figure 1. Pairwise comparisons versus day 0: *p < 0.05; **p < 0.01.

The clinical score significantly improved in the supplement group (by 14% by day 90, *p* < 0.0001) but not in the placebo group (11% improvement, *p* = 0.0692). The improvement in the supplement group was significant as of day 60 (*p* = 0.0426 and *p* = 0.0014 on days 60 and 90 respectively, Figure 2A; Table 3).

The data from each subscore (Figures 2B–E; Supplementary Table 1) showed that the effect in the supplement group mainly came from the pain upon palpation which was significantly improved over time (*p* < 0.0001) and as of day 60 in this group (*p* = 0.0152 and *p* = 0.0032 on days 60 and 90 respectively, Figure 2D; Supplementary Table 1).

### 3.4 Rescue analgesia

For ethical reasons, dogs were allowed to receive rescue analgesia if stopped at least one week before the next visit. Five dogs in the supplement group needed rescue analgesia once during the study for one to ten days. Another one needed it twice during the study for seven days each time. Three dogs in the placebo group received rescue analgesia once during the study, for one to seven days. The frequency of rescue analgesia was not significantly different between groups.

## 4 Discussion

This study evaluated the efficacy of a joint supplement for dogs containing a mixture of five main ingredients: ESM, hyaluronic acid (HA of different molecular weight), krill meal [rich in omega-3 fatty acids (42)], a natural source of astaxanthin [*Haematococcus pluvialis* (38)], and a *Boswellia serrata* extract. The results of this placebo-controlled study showed that all five main parameters assessed (LOAD, CBPI pain severity, CBPI pain interference, discomfort and clinical score) were significantly improved with the supplement while only the LOAD and CBPI pain interference were improved in the placebo group. Furthermore, the improvement of the latter parameter was significantly higher in the supplement group than in the placebo group. The test supplement is therefore efficacious in improving mobility and discomfort in osteoarthritic dogs.

A previous placebo-controlled pilot study with a similar formula as the one tested here (but without krill and low molecular weight HA) had shown promising results based on the LOAD score and inflammatory biomarkers like IL2 (61). This previous study had a lower number of animals per group and some parameters like the CBPI scores were not improved. In the present study, all main parameters assessed, including the CBPI and LOAD were improved. It is still unclear why the CBPI scores were not significantly improved in the preliminary study (61). The addition of krill, rich in omega-3 fatty acids (42–44), to the tested formula could be a potential explanation but it could also be due to a type II error in the previous study (61) or to other unexplained factors. The LOAD and CBPI, two main outcome measures in this study, are validated client-reported outcomes measures (CROMs – or clinical metrology instruments – CMIs) for the assessment of OA in dogs that have been used in many studies and are known to correlate well (15, 17, 61, 65–70). The CBPI assesses the magnitude of the pain over the last seven days (pain severity) and its impact on the dog’s mobility and daily activities like standing, walking, running or climbing up stairs (pain interference). The LOAD assesses the dog’s general mobility and mobility during exercise (no precision on the type of exercise), focusing on the level of activity and willingness to exercise, and on lameness rather than on the joint function (13). It also includes questions about the effect of weather on the dog’s activity (17). The rating scales are also different since the CBPI is based on an 11-point numerical rating scale while the LOAD is based on a 5-point Likert scale. Such differences could explain the discrepancies in this study and others (62, 70, 71). The results and interpretation of different CROMs may also vary depending on the dog population studied (breed, body weight, etc.), joint(s) affected, and components of the disease captured by the questionnaires, as outlined in previous studies (13, 65, 71). It is also possible that the owners have paid more attention to the way their dogs were moving over time, knowing which questions would be asked. This may explain partly the improvement observed in the placebo group for the CBPI pain interference and LOAD and could limit the conclusions of the study.

In the present study, we selected dogs with OA of the hip(s), elbow(s), tarsal or carpal joint(s) and excluded dogs with knee OA. Some dogs also had several joints affected. Despite the inclusion criteria to purposely limit the uncontrolled variability, the population selected varied in terms of joints affected (elbows, hips, tarsal joints or several of these). The proportion of joints affected did not differ significantly between groups. However, the OA grade and all assessed parameters tended to be worse in the supplement group than in the placebo group on day 0, with a significant difference between groups for the CBPI scores. This is opposed to what was found in the pilot study where dogs in the placebo group tended to be more severely affected (61). This difference, together with the differences in number of animals, formula and study population, could partly explain the differences in results observed between both studies.

The results obtained in discomfort and clinical scores, with significant differences in the supplement group but not in the placebo group, also support the beneficial action of the joint supplement. The effect would be particularly important on pain (as captured by the pain upon palpation and CBPI) rather than on the joint’s range of movement. This latter parameter may indeed depend on mechanical ones, like the presence of osteophytes and reduction of synovial space, that the supplement may not address as well. Other modalities, like activity modulation and rehabilitation, that may take longer to act, could also influence this parameter. A veterinary assessment was not performed in the pilot study but the change in some blood inflammatory markers like IL2 also suggested a positive evolution of the inflammatory process (61).

Based on the ingredients in the test supplement, an improvement in mobility is not surprising. Mobility improvements were also observed in studies assessing the effect of ESM in dogs with hip dysplasia or suboptimal joint function or in studies assessing the effect of *Boswellia serrata* in canine inflammatory joint and spinal disease (29, 34, 58). The omega-3 fatty acids (FA) found in krill may also play a part in this improvement, especially in the improvement of pain. Omega-3 FA have indeed been proven to have clinical analgesic efficacy in a meta-analysis (20). Studies have shown that amounts as low as 70 mg/kg body weight/day of eicosapentaenoic acid (EPA) and docosahexaenoic acid (DHA) from fish could decrease pain score and improve the quality of life of OA dogs (72, 73). However, the absorption and hence the efficacy of omega-3 FA also depends on the form and quality of these omega-3. It was found that the omega-3 from krill, bound to phospholipids, were better absorbed than those from other sources like fish or flaxseed, leading to a higher omega-3 index in dogs (42–44). These properties make krill a promising ingredient for joint disorders, including osteoarthritis, and other diseases (42, 45–48, 74, 75). The richness in phospholipids in krill can also help the absorption of other ingredients like astaxanthin – a potent antioxidant present in *Haematococcus pluvialis* and krill (38, 76) – and hyaluronic acid (50, 52). The addition of krill and HA of low molecular weight – for its known effect on cartilage maintenance (35, 36) – to the supplement already containing ESM, astaxanthin, a *Boswellia serrata* extract and hyaluronic acid of high molecular weight, has indeed given better results in dogs with mobility issues (60). The test supplement contains 1.86% of krill (around 74 mg) and therefore a lower dose of omega-3 FA than the one proven to be efficient in other studies. It is probably the combination of all the ingredients present in the test supplement and the synergy between them that make the test supplement efficient. The onset of action may depend on the severity of the disease, though. In dogs with light mobility disorders, owners felt an improvement in some mobility aspects in as early as seven days (59, 60). In this study, although some scores started to decrease by day 15, significant improvements were only observed by day 60 or 90, suggesting a longer onset of action in dogs with more severe OA. Importantly, all the ingredients present in the test supplement, from natural origin, are known to be safe and the good tolerance observed in this study and previous ones confirms it (59–61). The acceptability of the test supplement was also very good, allowing good compliance.

The significant difference observed between groups on day 0 for some parameters, despite the randomization, is the main limitation of the study. However, showing that all main parameters significantly improved with the supplement but not always with the placebo (or to a lesser extent) while the dogs were more severely affected in the former group, is a strong argument toward a beneficial action of the supplement. The lack of objective gait analysis is another limitation of this study that should be taken into account for future studies to limit the well-known placebo effect (13, 77). A previous study found that to detect a treatment effect in dogs with OA based on the CBPI, thresholds should be applied at inclusion (PIS and PSS ≥ 2) and success for each patient predefined as a decrease ≥ 1 in PSS and a decrease ≥ 2 in PIS (78). Success for each patient could be defined as a score decrease ≥ 4 for the LOAD (79). The current study design did not include these criteria due to their association with evaluating more severely impacted dogs and the need for an increased sample size, which would make recruitment especially challenging for a supplement trial. Furthermore, these criteria may be more applicable to test a drug and less so to test a supplement, which should not be given as a sole treatment for these advanced OA cases.

For the purpose of the study, we had to limit the treatments received by dogs but an efficient OA treatment cannot be based only on joint supplements and should include a variety of approaches including drugs (anti-inflammatory and analgesics) and physical therapy (18). However, efficient chondroprotective joint supplement like the one tested are still beneficial in OA dogs, regardless of the OA stage (1, 24).

## 5 Conclusion

This multicenter, double-blind, randomized, placebo-controlled study showed that the test supplement, containing ESM, krill meal, astaxanthin (via the algae meal *Haematococcus pluvialis*), hyaluronic acid and a *Boswellia serrata* extract, can improve the mobility and quality of life of dogs with OA. Indeed, all five main parameters tested (CBPI pain severity, CBPI pain interference, LOAD, discomfort, and clinical score) were significantly improved during the 90-day study in the group of dogs receiving the supplement while only two of them (CBPI pain interference and LOAD) improved in the placebo group and to a lesser extent. This study is in agreement with previous studies showing the effectiveness of the ingredients present in the test supplement in improving joint health. The test supplement could then be considered a valuable component of a multimodal approach to managing OA in dogs. Additionally, it may be beneficial for maintaining joint health, particularly in dogs predisposed to OA.

## Data Availability

The raw data supporting the conclusions of this article will be made available by the authors, without undue reservation.
