# Supplementary material for: Efficacy of a joint supplement containing eggshell membrane among other ingredients to improve the mobility of dogs with osteoarthritis: a multicenter double-blind randomized placebo-controlled study
Source: Front Vet Sci. 2025 Jun 2;12:1561793. doi: 10.3389/fvets.2025.1561793 (PMC12171436; doi:10.3389/fvets.2025.1561793)
Supplement: Supplementary file 1 [file Table_1.docx]

Supplementary Material

# Supplementary Table

**Supp Table 1:** Evolution of sub-parameters over time. Results are expressed as median (range). A lower score means better mobility, except for the quality of life (the higher, the better).

| **Assessment** | **Product** | **Day 0** | **Day 15** | **Day 30** | **Day 60** | **Day 90** | **p-value^a^** |
| --- | --- | --- | --- | --- | --- | --- | --- |
| CBPI Total | S | 11.37 (15.92) | 9.71 (15.58) | 8.67 (14.83) | 5.08 (14.67) ** | 4.12 (14.50) **** | 0.00001 |
|  | P | 6.62 (13.33) | 6.75 (14.00) | 4.83 (15.50) | 5.29 (17.67 * | 4.33 (16.92) * | 0.0186 # |
| CBPI Quality of life | S | 2 (3) | 2.5 (3) | 2.5 (3) | 3 (2) * | 3.5 (2) *** | 0.000007 |
|  | P | 3 (2) | 4 (3) | 3 (4) | 4 (3) | 4 (3) * | 0.0071 |
| Effect on Static Posture | S | 2.5 (3) |  | 2 (3) | 2 (3) | 2 (3) | 0.2104 |
|  | P | 2 (3) |  | 2 (3) | 2 (3) | 2 (3) | 0.0453 |
| Effect on Motion | S | 3 (3) |  | 3 (3) | 2 (3) | 2 (3) | 0.0038 |
|  | P | 3 (2) |  | 2 (2) | 2 (3) | 2 (3) | 0.0237 |
| Pain upon palpation | S | 3 (3) |  | 3 (3) | 2 (3) * | 2 (2) ** | 0.0000007 |
|  | P | 2 (3) |  | 2 (2) | 2 (3) | 2 (3) | 0.0062 |
| Passive ROM | S | 3 (3) |  | 3 (3) | 3 (3) | 3 (3) | 0.0875 |
|  | P | 2 (3) |  | 2 (3) | 2 (3) | 2 (3) | 0.6659 |

S: test supplement; P: placebo. a: p-value from the Friedman test. *: p<0.05; **: p<0.01; ***: p<0.001;****: p<0.0001 vs day 0 in the same group. #: p<0.05 when comparing groups between them.
